# First-in-class intranasal epinephrine spray for anaphylaxis: Dose finding clinical study

**DOI:** 10.1016/j.jacig.2025.100487

**Published:** 2025-04-22

**Authors:** Tair Lapidot, Yuval Tal, Dalia Megiddo, Galia Temtsin Krayz, Carolina Abrutzky, Simcha Blotnick, Oded Shamriz, Alon Hershko, Yoseph Caraco

**Affiliations:** a Nasus Pharma Ltd, Tel Aviv, Israel; b Allergy and Clinical Immunology Unit, Hadassah Medical Center, Faculty of Medicine, Hebrew University of Jerusalem, Jerusalem, Israel; d Clinical Pharmacology Unit, Hadassah Medical Center, Faculty of Medicine, Hebrew University of Jerusalem, Jerusalem, Israel; e Department of Internal Medicine, Hadassah Medical Center, Faculty of Medicine, Hebrew University of Jerusalem, Jerusalem, Israel; c Formulex Pharma Innovation Ltd, Nes Ziona, Israel

**Keywords:** Anaphylaxis, allergy, intranasal, bioavailability, epinephrine, powder, spray

## Abstract

**Background:**

Anaphylaxis is a life-threatening clinical presentation of acute systemic allergic reactions. Timely administration of epinephrine, usually by intramuscular autoinjector, is a robust life-saving treatment. Despite the critical necessity, there are multiple deterrents to patients’ proper use of epinephrine autoinjectors. FMXIN002 is a novel nasal dry powder formulation of epinephrine in a single-use device, offering first-in-class alternative treatment.

**Objectives:**

We sought to measure epinephrine pharmacokinetics, pharmacodynamics, and safety following a single administration of FMXIN002 at doses of 3.6 and 4.0 mg epinephrine versus intramuscular (IM) autoinjector 0.3 mg, in healthy adults.

**Methods:**

This was an open-label, single-dose, 3-treatment, crossover, randomized, comparative bioavailability study with 12 healthy adults, female and male. FMXIN002 stability was also tested.

**Results:**

FMXIN002 4.0 mg was absorbed faster and in higher amounts by most of the subjects, compared to IM autoinjector: 91% of subjects achieved the clinical threshold of 100 pg/mL plasma epinephrine at 6 minutes after administration of FMXIN002 4.0 mg compared to 55% of subjects treated with IM autoinjector. The area under the curve for 0 to 4 minutes’ period was significantly higher for FMXIN002 4.0 mg (geometric mean: 7.49 h ∙ pg/mL vs 2.06 h ∙ pg/mL, respectively; *P* = .0377). The pharmacodynamic response and safety were comparable among all treatments. No serious adverse events occurred, all events were mild and self-resolved. FMXIN002 was highly stable at all tested conditions including 5 years at 20 ± 5ºC.

**Conclusions:**

FMXIN002 4.0 mg nasal spray enables faster and higher epinephrine plasma absorbance at the short therapeutic window required for the treatment of anaphylaxis, using a patient-friendly, needle-free, stable and safe device.

Anaphylaxis is a life-threatening clinical presentation of acute systemic immediate allergic reactions. Prompt administration of epinephrine, usually by intramuscular (IM) autoinjector, is the life-saving treatment of choice for anaphylaxis, with a high rate of success when timely administered.^1^^,^^2^ Global anaphylaxis incidence is reported to be 50 to 112 episodes per 100,000 person-years. The rate of recurrence of anaphylaxis in high-risk patients is estimated to be 26.5% to 54.0%.^1^ Despite the critical clinical necessity, there are multiple deterrents to proper use of epinephrine autoinjectors by patients, such as fear of needles, short shelf life, high cost, and the inconvenience of carrying a cumbersome package. A recent survey conducted in the United States revealed that only 52% of adults with severe food allergy have been prescribed an autoinjector and 36% of adults believe the autoinjector can cause life-threatening side effects. Only 33% of the adults in the survey reported having an unexpired autoinjector.^3^ Adolescence is the period of highest risk of death from anaphylaxis.^4^^,^^5^ Nevertheless, Dupuis et al^5^ documented adolescents' actual autoinjectors adherence rate as 53% of the time, at most.

Efforts to develop alternative treatment modalities are ongoing.^6^ The first nasal liquid spray of epinephrine was recently approved in the United States and Europe.^7^^,^^8^ We have previously published the clinical results of a novel nasal inhaler based on dry powder formulation of epinephrine, FMXIN002.^9^ Dry powder has the advantages of long shelf life and better distribution in the nasal cavity, as compared to liquid form and hence quicker and higher absorption from the nose to the blood.^10^, ^11^, ^12^ These characteristics make powder epinephrine most appropriate to treat anaphylaxis due to its known short therapeutic window. FMXIN002 was successfully examined in a previously published clinical trial among 12 adults with a history of allergic rhinitis, under normal conditions and nasal congestion conditions following intranasal administration of an allergen, (Bioavailability of Nasal Epinephrine; NCT04696822).^9^ In that study, we used a maximum of 3.2 mg of epinephrine and observed that nasal congestion enhanced the absorption of epinephrine through the nasal mucosa. In the current study, we have conducted a dose optimization comparison under normal conditions, with a slightly higher concentration of epinephrine, aiming to select the optimal clinical dose of FMXIN002. Herein, we measured epinephrine pharmacokinetics, pharmacodynamics, and safety following a single administration of our powder-based nasal spray at doses of 3.6 and 4.0 mg versus IM autoinjector (EpiPen 0.3 mg; Viatris, Mylan Inc, Canonsburg, Pa), in healthy adults.

## Methods

### Study participants and design

This was an open-label, single-dose, 3-treatment (A-C), crossover, randomized, 2-sequence, comparative bioavailability study. Healthy, nonsmoking male and nonpregnant or nonlactating female volunteers, aged 18 to 55 years, with a body mass index between 18 and 30 kg/m^2^, were enrolled and screened. Exclusion criteria included the presence of a medical condition requiring regular medication (prescription or over-the-counter) with systemic absorption other than oral contraceptives. The screening procedure included a nasal cavity examination. Eligible subjects, 6 per group, were randomized to 1 of the 2 sequences.

**Fig 1:**
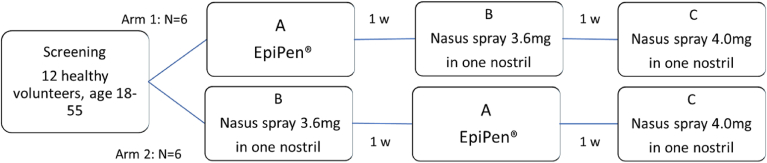
Study design. The subjects were randomly assigned to 1 of 2 sequences (arms 1 and 2), each of which consisted of 3 epinephrine administrations: 1 IM (treatment A, EpiPen 0.3 mg) and 2 escalating doses via intranasal route (treatment B, 3.6 mg; treatment C, 4.0 mg). The administrations were separated by 1 week of washout. Pharmacokinetics and vital signs were followed at each administration day from −1 hour before to 2 hours post dose administration. Safety was also followed by AEs, physical examination, 12-lead ECG, safety laboratory (hematology, chemistry, urinalysis) evaluations, nasal cavity examination, nasal and nonnasal symptoms questionnaire.

Treatment A was a single dose of the reference product EpiPen (0.3 mg) administered IM into the vastus lateralis. Treatment B was the nasal powder spray FMXIN002 epinephrine 3.6 mg, 1 actuation administered into the nostril. Treatment C was the nasal powder spray FMXIN002 epinephrine 4.0 mg, once into the nostril. There was a 1-week washout period between each dosing day (Fig 1).

Standardized meals were provided throughout each treatment day. Water consumption was restricted from 1 hour prior to drug administration to 1 hour post dosing. Access to water was otherwise freely available to the participants. The participants remained in a supine position for ≥1 hour following drug administration and then in a sitting or semireclined position for the next 3 hours, to enable measurements and as a safety precaution.

Clinical parameters assessments were recorded at screening, each dosing day, and at the end of the study period. They included vital signs, laboratory evaluation including hematology, chemistry, urinalysis, and 12-lead electrocardiogram (ECG). Blood pressure, heart rate temperature, and respiratory rate were monitored before dosing and every few minutes after dosing up to 4 hours later. A 12-lead ECG was recorded before dosing and 45 to 60 minutes after administration. Nasal cavity examination and scoring were done at screening, before and after each study drug administration, and at the end of the study (using a published nasal examination worksheet).^13^ Total symptom score^13^ of nasal and nonnasal symptoms (runny nose, nasal congestion, nasal itching, sneezing, watery eyes, itchy eyes, redness of eyes, itching of ears, itching of throat, and anosmia) was completed by the participant at screening, after each dosing and at end of study.^14^

Epinephrine pharmacokinetics was evaluated based on 13 plasma samples collected on each dosing day, including at −1-, −0.5-, 0-hour sampling before dose administration for endogenous epinephrine baseline measurement and at 2, 4, 6, 8, 10, 15, 20, and 30 minutes and 1 and 2 hours post dose administration.

The blood samples were placed in an ice bath and immediately separated by cold centrifugation frozen and stored at −80 ± 15°C until analysis. Epinephrine levels in plasma samples were measured using a validated liquid chromatography-tandem mass spectrometry method, conducted in accordance with Organization for Economic Cooperation and Development Good Laboratory Practice standards.

### Statistical analysis

Statistical analyses were performed on quality-assured data, using the PROC GLM procedure from SAS (version 9.4; SAS Institute, Cary, NC).

Mixed model, least squares means (LSM), and Dunnett comparisons were performed on log-transformed baseline-corrected area under the curve (AUC)_t_, AUC_inf_, AUC_0-4 min_, AUC_0-10 min_, AUC_0-30 min_, and the maximum concentration of the drug observed in the subjects' plasma following administration (C_max_).

Using the same statistical model, the LSM, the differences between the treatments LSM, and the corresponding SEs of these differences were estimated for log-transformed AUC_t_, AUC_inf_, AUC_0-4 min_, AUC_0-10 min_, AUC_0-20 min_, AUC_0-30 min_, and C_max_ parameters. Based on these statistics, the ratios of the geometric means for treatments and the corresponding 90% CI were calculated for comparisons.

The treatment differences in the time to reach C_max_ (T_max_), as well as T_50__pg/mL_, T_100__pg/mL_, T_200__pg/mL_ were evaluated by a nonparametric approach (Wilcoxon signed rank test) on the untransformed values.

The proportion of subjects who achieved certain epinephrine plasma concentrations (eg, 50, 100, and 200 pg/mL) were compared among the treatments.

### Ethical approval of the study

The study was performed at the Clinical Research Unit of Hadassah Medical Center (Jerusalem, Israel). The study protocol was approved by Hadassah’s Institutional Review Board (IRB number: 0206-23-HMO), and following a detailed explanation, the participants signed an informed consent (registered at ClinicalTrials.gov as NCT06205134 and at https://my.health.gov.il/CliniTrials/Pages/MOH_2023-07-01_012776.aspx).

### FMXIN002 preparation and stability testing

The epinephrine powder was manufactured (Formulex Pharma Innovation, Nes Ziona, Israel) and filled into disposable Unit Dose Powder Devices (UDS, Aptar Pharma, France). The drug-device product FMXIN002 was analyzed to control the powder particle size, drug content, shot weight, purity, uniformity, microbial cleanliness, and stability. Each FMXIN002 was packed in a sealed protective pouch and contained 3.6 mg or 4.0 mg epinephrine. The clinical batch was successfully produced in compliance with Good Manufacturing Practice. The stability of FMXIN002 was evaluated at various storage conditions for 5 years at 20 ± 5°C conditions without humidity control, 2 years at 20 ± 5°C at40% relative humidity (RH) and 6 months at accelerated conditions 40°C at75% RH.^15^

## Results

### Clinical study subject demographics

Twelve healthy Caucasian adults, 50% of which were female, were included in the study. The mean age was 26.1 ± 5.8 years, mean height 166.8 ± 10.6 cm, mean weight 68.4 ± 13.9 kg and mean body mass index 24.4 ± 2.7 kg/m^2^ (Table I). All the enrolled subjects were healthy according to the inclusion and exclusion criteria. One subject withdrew from the study before participating in treatment C due to travel abroad, for a reason not related to the study.

**Table I:** Subject baseline demographics

Parameter	Study population (N = 12)
Age (y)	
Mean ± SD	26.1 ± 5.8
Median (min, max)	23.0 (20.0, 35.0)
Gender, n (%)	
Female	6 (50.0)
Male	6 (50.0)
Race, n (%)	
Caucasian	12 (100)
Ethnicity, n (%)	
Not Hispanic or Latino	12 (100)
Height (cm)	
Mean ± SD	166.8 ± 10.6
Median (min, max)	162.0 (154.0, 190.0)
Weight (kg)	
Mean ± SD	68.4 ± 13.9
Median (min, max)	64.0 (53.0, 98.0)
BMI (kg/m^2^)	
Mean ± SD	24.4 ± 2.7
Median (min, max)	24.2 (20.2, 29.6)

*BMI,* Body mass index.

**Table II:** Plasma pharmacokinetic parameters of investigational and reference treatments (after baseline subtraction)

Parameter	Treatment	n	Arithmetic mean (CV)∗	Geometric mean	(95% CI)	Comparison	*P* value differences of LSM	Signed rank test	*P* value type 3 tests of fixed effects
C_4 min_ (pg/mL)	A	12	164.7 (135.4)	61.24	25.66-146.15	A vs B	.2325	0.2402	.1985
						A vs C	.0798	0.1934	
	B	12	371.7 (139.8)	140.96	58.00-342.60				
	C	11	504.5 (113.8)	202.62	65.27-628.97				
C_10 min_ (pg/mL)	A	12	263.6 (87.8)	181.32	112.68-291.76	A vs B	.4576	0.4697	.7358
						A vs C	.5802	0.8311	
	B	12	352.1 (122.4)	174.14	82.18-368.97				
	C	11	343.8 (70.6)	231.91	113.85-472.39				
C_20 min_ (pg/mL)	A	12	251.6 (61.7)	200.07	133.73-299.36	A vs B	.9269	0.5186	.9818
						A vs C	.9196	0.8311	
	B	12	242.9 (151.7)	128.87	72.22-229.96				
	C	11	267.5 (61.7)	223.41	154.86-322.29				
C_max_ (pg/mL)	A	12	427.0 (51.4)	360.48	255.30-508.97	A vs B	.7208	0.6772	.3001
						A vs C	.1393	0.5195	
	B	12	497.9 (100.0)	338.40	209.84-545.72				
	C	11	730.4 (111.3)	477.02	289.83-785.10				
T_max_ (h)∗	A	12	0.3 (0.1-0.5)	0.25	0.17-0.36				
	B	12	0.1 (0.0-2.0)	0.15	0.08-0.28				
	C	11	0.2 (0.0-2.0)	0.17	0.08-0.35				
AUC_0-4 min_	A	12	5.8 (149.3)	2.06	0.90-4.72	A vs B	.3929	0.3804	.1075
(h ∙ pg/mL)						A vs C	.0377	0.2061	
	B	12	14.2 (135.0)	5.33	2.31-12.28				
	C	11	27.4 (138.9)	7.49	2.08-27.03				
AUC_0-10 min_	A	12	27.4 (103.4)	16.09	8.85-29.28	A vs B	.2720	0.2036	.2601
(h ∙ pg/mL)						A vs C	.1131	0.1748	
	B	12	51.0 (122.1)	23.40	10.52-52.06				
	C	11	64.0 (101.1)	33.16	13.78-79.80				
AUC_0-20 min_	A	12	71.0 (78.7)	54.17	36.25-80.94	A vs B	.4473	0.3804	.5787
(h ∙ pg/mL)						A vs C	.3251	0.5195	
	B	12	99.2 (126.2)	52.24	26.58-102.69				
	C	11	111.4 (83.0)	76.79	42.26-139.53				
AUC_0-30 min_	A	12	118.8 (61.0)	98.85	70.01-139.57	A vs B	.7583	0.9097	.8094
(h ∙ pg/mL)						A vs C	.5205	0.7646	
	B	12	133.9 (131.0)	75.82	42.10-136.56				
	C	11	153.9 (71.7)	120.47	77.18-188.03				
AUC_t_	A	12	360.5 (48.0)	315.17	233.64-425.14	A vs B	.9722	0.5186	.6612
(h ∙ pg/mL)						A vs C	.4212	0.2061	
	B	12	363.6 (97.2)	289.25	210.04-398.33				
	C	11	434.9 (42.5)	399.74	314.35-508.33				
AUC_inf_	A	6	400.6 (45.5)	360.31	232.99-557.20	A vs B	.0037	0.5000	.0072
(h ∙ pg/mL)						A vs C	.1117	0.5000	
	B	2	1731.5 (6.2)	1729.85	1312-2282				
	C	4	670.4 (43.3)	624.69	375.13-1040				
AUC_t_/AUC_inf_ (%)	A	6	87.9 (10.5)	87.48	79.99-95.68				
	B	2	46.2 (104.6)	31.09	0.08-12535				
	C	4	63.4 (22.4)	62.18	47.77-80.93				
T_1/2_ (h)∗	A	6	0.6 (0.3-1.0)	0.57	0.41-0.79				
	B	2	5.6 (0.9-10.3)	2.99	0.00-7191				
	C	4	1.7 (0.8-2.2)	1.44	0.77-2.69				
Kel (1/h)	A	6	1.3 (40.0)	1.23	0.88-1.72				
	B	2	0.4 (199.4)	0.23	0.0-565.86				
	C	4	0.5 (53.7)	0.48	0.26-0.90				
T_LIN_ (h)	A	6	0.4 (43.7)	0.35	0.22-0.55				
	B	2	0.5 (0.0)	0.50	0.50-0.50				
	C	4	0.3 (53.8)	0.30	0.14-0.64				
T_50 pg/mL_ (h)∗	A	12	0.100 (0.067-0.167)						
	B	12	0.067 (0.033-0.500)						
	C	11	0.067 (0.033-0.033)						
T_100 pg/mL_ (h)∗	A	11	0.167 (0.067-0.500)						
	B	11	0.067 (0.033-2.000)						
	C	11	0.067 (0.033-0.500)						
T_200 pg/mL_ (h)∗	A	10	0.117 (0.033-0.333)						
	B	8	0.050 (0.033-0.100)						
	C	9	0.033 (0.033-0.133)						

*C*_*4 min*_*/C*_*10 min*_*/C*_*20 min*_, measured concentration at 4/10/20 minutes post dose; *CV,* coefficient of variation; *Kel,* elimination rate constant; *T*_*50 pg/mL*_*/T*_*100 pg/mL*_*/T*_*200 pg/m*_*,* time when epinephrine concentration is 50/100/200 pg/mL; *T*_*LIN*_*,* start time for linear regression.

∗ For T_max_, T_50__pg/mL_, T_100__pg/mL_, and T_200__pg/mL_, median (min, max) is shown.

### Pharmacokinetic effect and statistical analysis results

**Fig 2:**
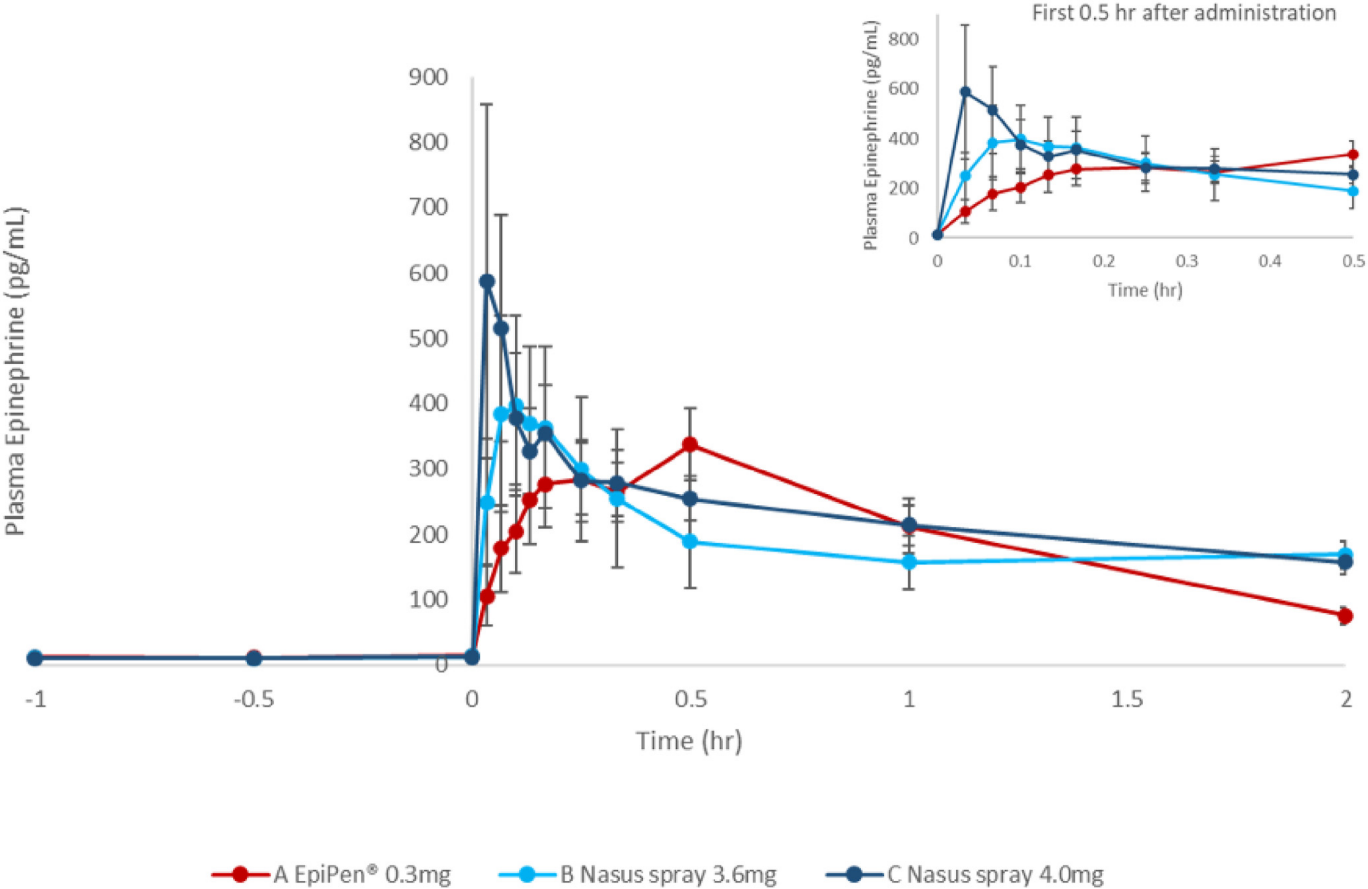
Mean measured plasma epinephrine concentration time profile at −1 to 2 hours. Inset shows mean measured plasma epinephrine concentration at 0 to 0.5 hours. **(A)** EpiPen 0.3 mg, **(B)** Nasus spray 3.6 mg, **(C)** Nasus spray 4.0 mg. Results are mean ± SE.

Plasma pharmacokinetic parameters of investigational and reference treatments are summarized in Table II. Mean plasma epinephrine at 2-hour concentration time profiles and 0.5 hours are shown in Fig 2.

**Fig. 3:**
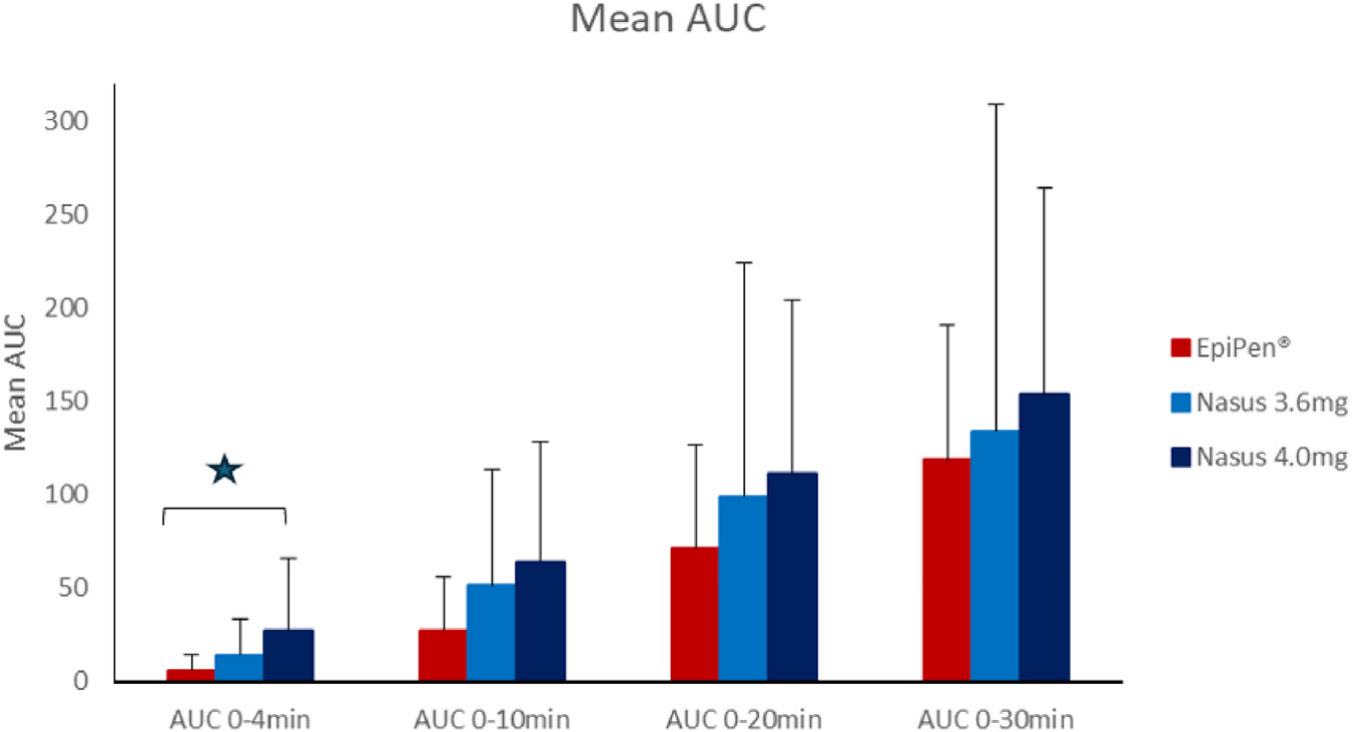
Comparison of mean AUC (baseline-subtracted). ∗*P* = .037 by differences of LSM.

Among the 3 treatments administered, FMXIN002 4.0 mg demonstrated the highest C_max_ and the highest mean AUC for up to 30 minutes post administration, followed by FMXIN002 3.6 mg, with EpiPen having the lowest C_max_ and AUC for the first 30 minutes post administration (Table II and Fig 3). AUC_0-4 min_ was statistically significantly higher for FMXIN002 4.0 mg compared to EpiPen (geometric mean: 7.49 h ∙ pg/mL [95% CI: 2.08-27.03] vs 2.06 h ∙ pg/mL [95% CI: 0.90-4.72]; *P=* .0377) (I). The concentration of FMXIN002 4.0 mg at 4 minutes post administration (C_4min_) was the highest among the 3 treatments with a trend for a statistically significant difference compared to EpiPen (202.62 pg/mL [95% CI: 65.27-628.97] vs 61.24 pg/mL [95% CI: 25.66-146.15]; *P* = .0798) (Table II).

The concentration of FMXIN002 4.0 mg was higher at all other time points (10, 30 minutes) but did not reach statistical significance.

**Fig 4:**
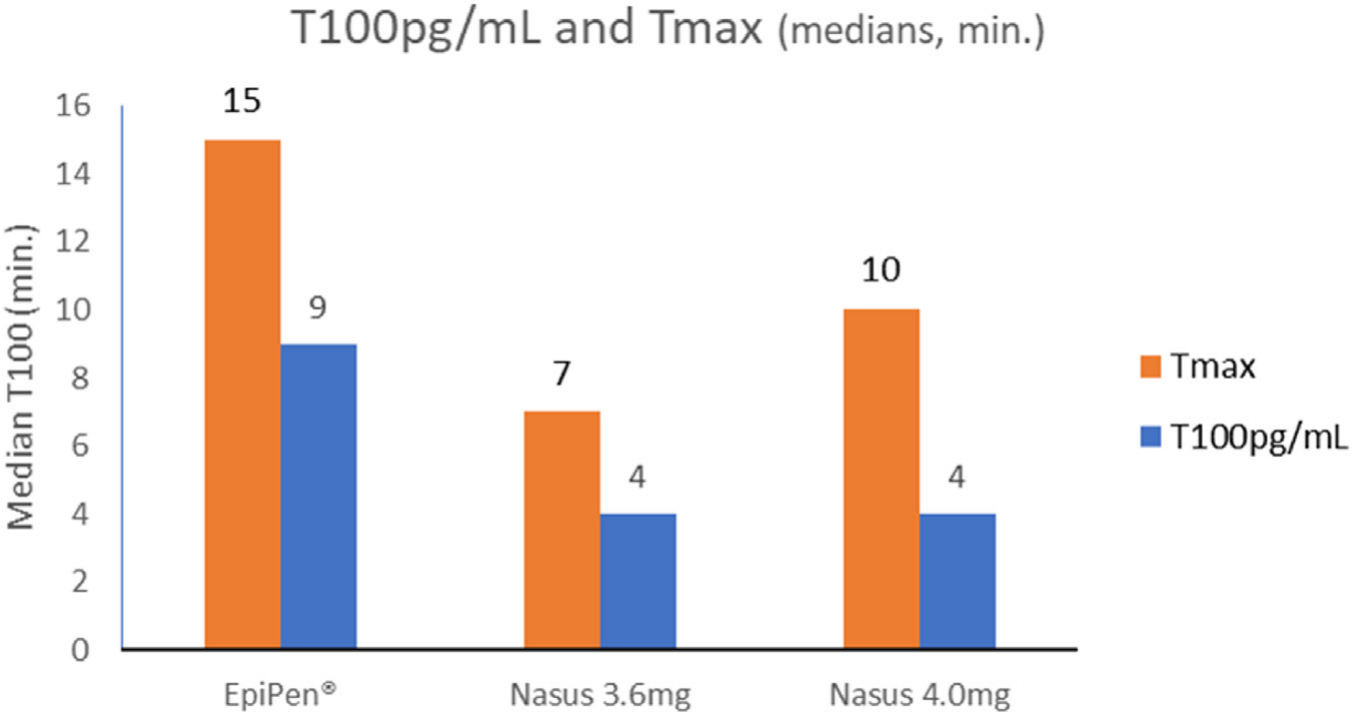
Median time (minutes) results: T_100__pg/mL_ and T_max_. T_max_ is the time to reach the maximum concentration of the drug observed in the plasma following administration. T_100__pg/mL_*is the t*ime when epinephrine concentration in plasma is 100 pg/mL (clinical threshold).

FMXIN002 3.6 mg demonstrated the shortest median T_max_ (7 minutes) compared to FMXIN002 4.0 mg (10 minutes) and EpiPen (15 minutes) (Table II and Fig 4).

Both FMXIN002 3.6 mg and FMXIN002 4.0 mg showed a concentration of 100 pg/mL (ie, the clinical threshold) after a median of 4 minutes compared to a median of 9 minutes for EpiPen (Table II and Fig 4).

**Fig 5:**
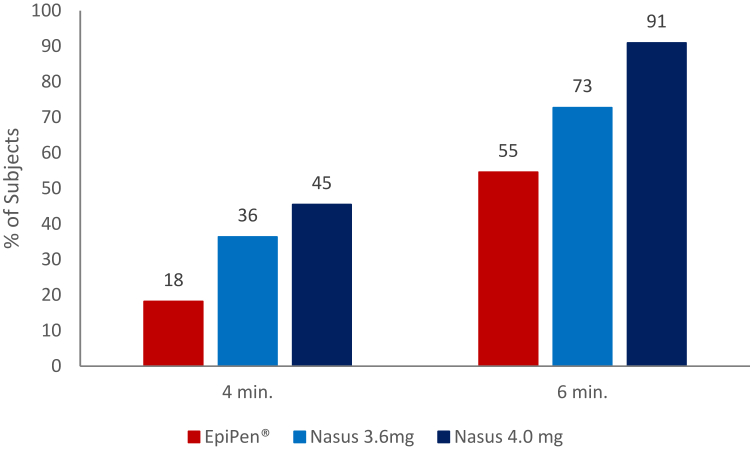
Proportion of subjects achieving clinical threshold of 100 pg/mL at 4 and 6 minutes.

Ten subjects (91%) treated with FMXIN002 4.0 mg achieved the clinical threshold of 100 pg/mL during the first 6 minutes after administration compared to 8 subjects (73%) treated with FMXIN002 3.6 mg and 7 subjects (55%) treated with EpiPen (Fig 5). At 10 minutes after administration, 81% of the subjects treated with FMXIN002 4.0 mg achieved plasma epinephrine of ≥200 pg/mL compared to only 33% after the treatment with EpiPen.

### Pharmacodynamic results

**Fig 6:**
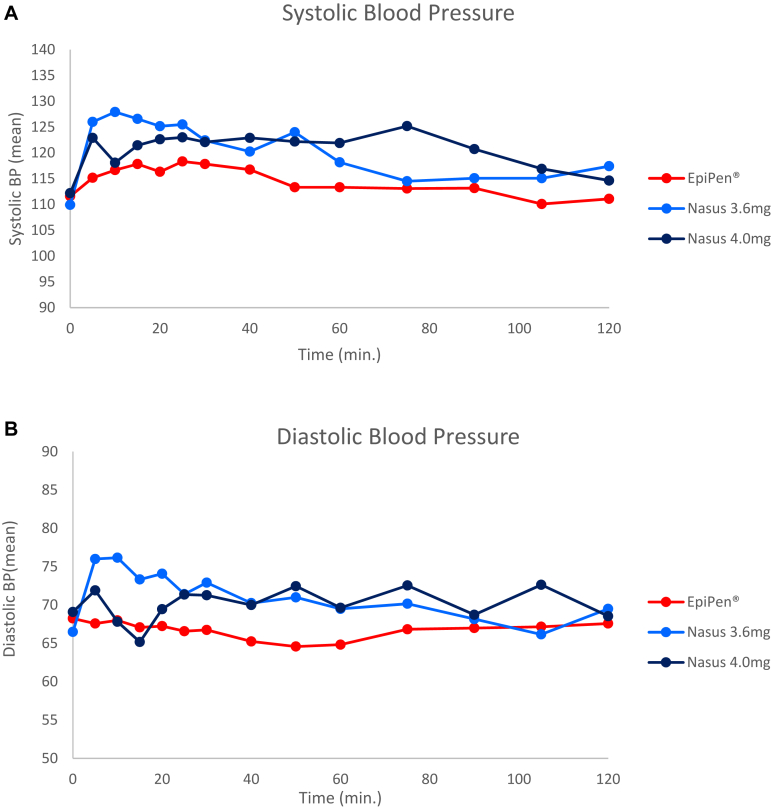

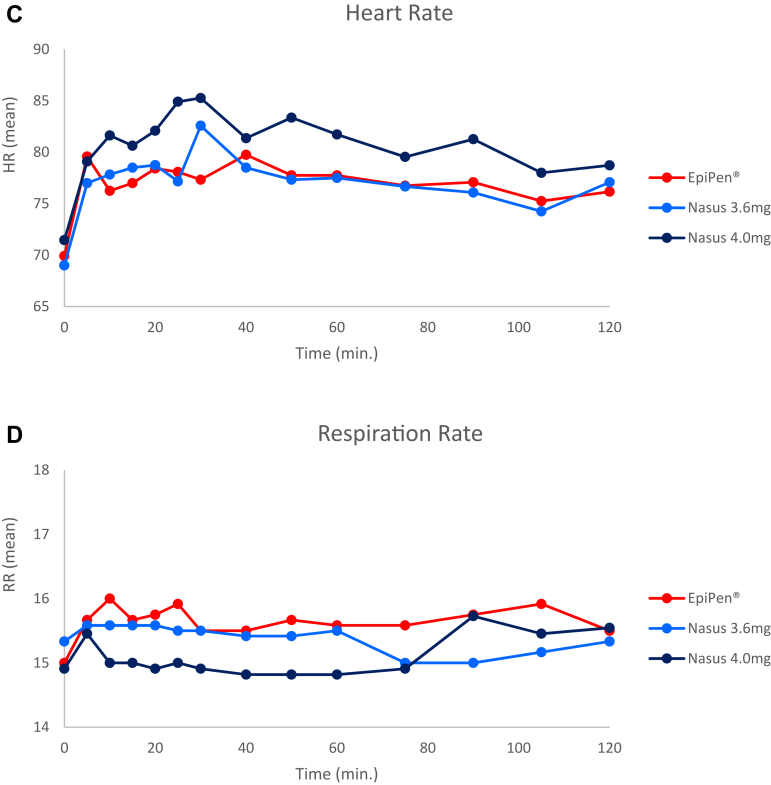
Pharmacodynamic parameters at 0 to 120 minutes post dose. **(A)** Systolic blood pressure (BP), **(B)** diastolic BP, **(C)** heart rate (HR), **(D)** respiratory rate (RR). All results are presented as means. There were no significant differences compared to EpiPen. All results were within normal clinical ranges. (SE is not presented for graphical clarity.)

Following all treatments, systolic blood pressure, mean heart rate, and respiratory rate slightly increased right after dosing and returned to normal by 2 hours post dosing. The mean blood pressure response was higher after the nasal spray in comparison to EpiPen; however, all the measured values of all vital signs remained within normal clinical range and no statistical difference between the treatments was observed (Fig 6).

### Safety

A total of 24 adverse events (AEs) were reported by 9 subjects (75%). Eighteen events in 6 subjects (50.0%) were considered related to the study treatment. All events were mild, transient and self-resolved. No serious AEs occurred. None of the subjects withdrew from the study due to an AE. More events related to epinephrine effect (palpitations, headache) were reported after the nasal administration; however, there were no statistically significant differences in the rates of AEs (overall and treatment-related) among the treatment groups. A summary of treatment-related AEs is presented in Table III. No clinically significant abnormal physical examination results, including nasal cavity examination findings, were reported. No clinically significant changes in laboratory test values (hematology, chemistry, and urinalysis) were observed between screening and the end of the study. No clinically significant abnormal findings were noted in physical examination, vital signs, or ECG results.

**Table III:** Summary of treatment-related AEs by MedDRA system organ class and preferred term

System organ class/preferred term	EpiPen (n = 12)		FMXIN002 3.6 mg (n = 12)		FMXIN002 4.0 mg (n = 11)	
Subjects with ≥1treatment-related∗ AE	2	(16.7)	4	(33.3)	4	(36.4)
Cardiac disorders						
Palpitations	1	(8.3)	1	(8.3)	3	(27.3)
Eye disorders						
Eye pain	0	(0)	1	(8.3)	0	(0)
Gastrointestinal disorders						
Nausea	0	(0)	1	(8.3)	0	(0)
General disorders and administration site conditions					0	(0)
Asthenia	0	(0)	1	(8.3)	0	(0)
Investigations						
Blood pressure increased	0	(0)	2	16.7	0	(0)
Nervous system disorders						
Headache	0	(0)	3	(25.0)	2	(18.2)
Tremor	1	(8.3)	0	(0)	0	(0)
Respiratory, thoracic, and mediastinal disorders						
Productive cough	0	(0)	0	(0)	1	(9.1)
Skin and subcutaneous tissue disorders					1	(9.1)
Pruritus	0	(0)	0	(0)	1	(9.1)

*MedDRA*, Medical Dictionary for Regulatory Activities.

Values are n (%).

∗ All treatment-related AEs were considered “probably related.”

Symptoms were reported in the nasal and nonnasal questionnaire after dosing. Four subjects experienced symptoms after dosing with the nasal spray (3.6 mg and 4.0 mg). The most common was runny nose and sneezing. All symptoms were transient and resolved without treatment. There were no reports of anosmia.

### Stability results

FMXIN002 products were highly stable at all tested conditions: 5 years at 20 ± 5°C conditions (stored without humidity control), 2 years at 20 ± 5°C at 40% RH, and 6 months at accelerated conditions (40°C at 75% RH).

## Discussion

The goal of this study was to assess the safety and comparative bioavailability and pharmacodynamic response between a single dose of epinephrine microspheres powder nasal inhaler FMXIN002 and epinephrine injection, USP auto-injector 0.3 mg for IM injection in healthy adults (EpiPen).

Building on the findings of our previous study involving volunteers with and without nasal congestion, where nasal epinephrine doses of ≤3.2 mg were administered,^9^ we cautiously increased the dose to 3.6 and 4.0 mg in this study. The goal was to achieve adequate plasma epinephrine levels in all participants, including those who may not experience nasal congestion during a severe allergic reaction.

FMXIN002 4.0 mg was absorbed significantly faster and more efficiently than EpiPen. The results demonstrated higher mean C_max_, and higher mean AUC during the first 30 minutes after treatment compared to the reference drug, EpiPen. AUC for the first 4 minutes was 4.7-fold higher following FMXIN002 4.0 (27.44 h ∙ pg/mL) versus AUC after EpiPen (5.85 h ∙ pg/mL). This difference was statistically significant despite the rather small sample size of 12 subjects. FMXIN002 4.0 mg also showed a shorter median T_max_ and T_100__pg/mL_ than EpiPen. The total exposure over 2 hours (AUC_t_) was comparable to that of EpiPen.

Both FMXIN002 3.6 mg and 4.0 mg showed a concentration of 100 pg/mL (ie, the clinical threshold of epinephrine effect)^16^, ^17^, ^18^ after a median of only 4 minutes compared to a median of 9 minutes for EpiPen. Moreover, the proportion of subjects who achieved the clinical threshold fast was much higher after the nasal spray. Ten subjects (90.91%) treated with FMXIN002 4.0 mg achieved the clinical threshold of 100 pg/mL during the first 6 minutes after administration compared to 8 subjects (72.73%) treated with FMXIN002 3.6 mg and 7 subjects (54.55%) treated with EpiPen. At 10 minutes after administration, 81% of the subjects treated with FMXIN002 4.0 mg achieved plasma epinephrine of ≥200 pg/mL, 58% after FMXIN002 3.6 mg, while 67% failed to reach 200 pg/mL after the autoinjector.

FMXIN002 was well tolerated at both doses tested. All treatment-emergent AEs were mild and transient. No severe AEs, serious AEs, or deaths occurred during the study. No subject withdrew from the study due to AEs.

There were no statistically significant differences in the rates of AEs (overall and treatment-related) among the treatment groups. No clinically significant changes in laboratory values or ECG over time were observed. Following administration of the reference and test products, mean heart rate, respiration rate, and systolic blood pressure increased similarly and returned near baseline within 2 hours. The diastolic blood pressure after FMXIN002 was slightly higher than after the autoinjector. The measured values were within normal clinical range and there was no statistical difference between the treatments.

Altogether the analyses indicate that FMXIN002 4.0 mg is absorbed faster by most of the subjects and achieves the clinical threshold of 100 pg/mL plasma epinephrine faster than EpiPen, with a similar pharmacodynamic response, safety profile, and total exposure level. The 4.0 mg dose was therefore selected for further development.

These fast absorptions of epinephrine through Nasus dry powder formulation demonstrated by short T_max_ and significantly higher AUC at the first minutes (median T_max_: 10 minutes; AUC_0-10 min_, geometric mean: 33.16 h ∙ pg/mL), are significantly higher and faster than the reported for the recently approved liquid nasal epinephrine spray (median T_max_: 30 minutes; AUC_0-10 min_, geometric mean: 11.7 h ∙pg/mL)^19^ and other in-development liquid-based nasal epinephrine (median T_max_: 20.5 minutes: AUC_0-10 min_, geometric mean: 18.8 h ∙ pg/mL.^19^^,^^20^ The absorption results following FMXIN002 4.0 mg administration are also faster and higher than the results obtained after sublingual administration of 12 mg epinephrine to healthy volunteers (median T_max_: 12 minutes; AUC_0-10 min_, geometric mean: 11.0 h ∙ pg/mL).^20^

The enhanced bioavailability of FMXIN002 is based on the well-described phenomena of the wider distribution of dry powder in the nasal cavity compared to liquid nasal formulations.^11^^,^^12^^,^^21^^,^^22^ Williams et al^23^ have reported by *in vitro* human nasal cast and *in vivo* studies in healthy volunteers that significantly higher deposition (*P* < .05) of a radiolabeled study molecule was observed in the olfactory region for the unit-dose powder device compared to the multidose liquid nasal spray pump. In fact, 64% of the deposited dose in the liquid spray was found in the nose and lower regions of the nasal cavity, where drug absorption into the blood is less effective, while only 6% was measured in the olfactory region, where the absorption is effective. In contrast, after powder spray, 34% of the drug was observed in the olfactory region.^12^

Another important advantage of dry powder formulation is a longer stability period in various climate conditions. Epinephrine is a sensitive molecule in liquid formulations and undergoes oxidation and degradation to a nonactive molecule. As a result, liquid forms of epinephrine have limited shelf life despite added stabilizers and are not recommended for use if exposed to extreme climate conditions.^24^^,^^25^ However, epinephrine in dry powder form is stable at room temperature without added preservatives. FMXIN002 was found stable for 5 years at room conditions without humidity control, as well as in standard stability testing of 2 years at 20 ± 5°C at 40% RH and 6 months under extreme conditions of heat and humidity. These attributes could well be meaningful and useful for the patients, caregivers, and health insurance payers.

The World Allergy Organization Anaphylaxis Committee and the World Allergy Organization Junior Members Steering Group recently published the results of a global survey to evaluate local practice in the diagnosis and management of anaphylaxis across regions. They have found that epinephrine autoinjectors are only available in 60% of countries surveyed, mainly in high-income countries. Many countries in South America, Africa/Middle-East, and Asian-Pacific regions do not have epinephrine autoinjectors available.^26^ We suggest that the high stability of our dry powder drug product under different climate conditions would encourage distribution around the world where autoinjectors are not yet available. Additionally, its compact size—just a few centimeters—may serve as a positive incentive for patients who are less consistent in carrying the bulky 2-autoinjector package at all times.

Our study has several limitations. Mainly, the small sample size and the demographic profile of our volunteers, who were all healthy at the age of 20 to 35 years. Further studies are needed to explore the response in other demographic groups and larger sample size. Nevertheless, the results demonstrate the potential of FMXIN002 powder-based nasal spray 4.0 mg epinephrine as a future treatment for anaphylaxis with the fastest plasma absorbance using a comfortable patient-friendly, needle-free, stable, and safe device.

## Disclosure statement

The study was funded by Nasus Pharma, Israel.

Disclosure of potential conflict of interest: D. Megiddo, C. Abrutzky, and T. Lapidot are employees of Nasus Pharma. Y. Tal is a consultant to Nasus Pharma. G.T. Krayz provides manufacturing and analytical services to Nasus Pharma. G. T. Krayz, D. Megiddo, C. Abrutzky, and T. Lapidot hold patents for the FMXIN002 nasal spray or are related to the patents assigned to Nasus Pharma. The rest of the authors declare that they have relevant conflicts of interest.
